# Knockdown of DIAPH3 Inhibits the Proliferation of Cervical Cancer Cells through Inactivating mTOR Signaling Pathway

**DOI:** 10.1155/2021/4228241

**Published:** 2021-10-06

**Authors:** Linling Wan, Jiamin Zhu, Qunying Wu

**Affiliations:** ^1^Department of Obstetrics and Gynecology, The Affiliated Jiangyin Hospital of Southeast University Medical College, Wuxi, Jiangsu 214000, China; ^2^Department of Oncology, The Affiliated Jiangyin Hospital of Southeast University Medical College, Wuxi, Jiangsu 214000, China

## Abstract

Cervical cancer (CC) ranks fourth for both incidence and mortality among females in worldwide. Therefore, it is urgent to explore new therapeutic and diagnostic targets for cervical cancer. Diaphanous-related formin 3 (DIAPH3) has been identified to play crucial roles in many malignant tumors. But its function and potential mechanism in CC remain largely unknown. In our study, DIAPH3 was frequently upregulated in CC tissue samples and increased expression of DIAPH3 was associated with poor overall survival according to several databases. Through *in vitro* and *in vivo* experiments, we found that decreased expression levels of DIAPH3 significantly inhibited the progression of CC. The GSEA analysis and western blot assay indicated that DIAPH3 was associated with the mTOR signaling pathway. The univariate and multivariate Cox analysis indicated that DIAPH3 was an independent prognosis risk factor in TCGA-CESC. And we confirmed that DIAPH3 expression was clearly related to tumor immune infiltrating cells (TIICs) by the analysis of CIBERSORT and TIMER databases. Taken together, we revealed that DIAPH3 plays as an oncogene through mTOR signaling pathway and DIAPH3 might be a potential prognostic biomarker in CC.

## 1. Introduction

Cervical cancer (CC) is a kind of common malignancies worldwide. And both the incidence and the mortality of CC remain fourth among females [1]. There is a substantial increase in cervical cancer incidence in China [2]. Most cases are often diagnosed at an advanced stage. Almost all cases are due to human papillomavirus (HPV) infection [3]. The treatments of CC include surgery or a concurrent chemoradiotherapy program composed of cisplatin-based chemotherapy with brachytherapy and external beam radiotherapy [4]. Despite the advancement of diagnosis and treatment technology, the five-year survival rates of advanced CC are about 30%–50% [5]. Hence, it is urgent to explore new therapeutic and diagnostic targets for cervical cancer.

The diaphanous-related formin 3 (DIAPH3) is considered a core modulator of the cytoskeleton [6] and the host gene locates on human chromosome 13q21.2 [7]. DIAPH3 is investigated to reshape the cytoskeleton [8] and regulates the expression of GSK3*β* to preserve microtubule stabilization [9]. Moreover, previous studies reported that DIAPH3 plays many crucial roles in many malignancies. Di Vizio D et al. confirmed that DIAPH3 facilitates the metastasis process of prostate cancer [10]. Jiang et al. verified that the overexpression of DIAPH3 suppresses the migration of triple-negative breast cancer [11]. Dong et al. reported that DIAPH3 facilitates the malignant biological behavior of HCC cells through activating *β*-catenin/TCF signaling pathway [12]. To our knowledge, the functions of DIAPH3 and its molecular mechanism in cervical cancer were largely unknown. In the present study, we revealed that DIAPH3 plays as an oncogene through mTOR signaling pathway. We revealed that DIAPH3 promoted proliferation through mTOR signaling pathway in cervical cancer.

In recent years, tumor immunity has gradually attracted the attention of many researchers. The regulatory T cells (T regs) could affect the role of cytotoxic T cells to facilitate tumor cells to get rid of the immune system [13]. A large number of papers have deeply explored the complex tumor immune regulation process and immune checkpoints to influence the process of antitumor immune response. For example, the advancement of antibody drugs against PD1 and PD-L1 for the therapy of prostate cancer and renal cell carcinoma has shown great efficacy in clinical practice [14–17]. Therefore, in this study, we further evaluated the prognostic value of DIAPH3 and assessed the association between the expression of DIAPH3 and tumor immune infiltration level in CC.

## 2. Materials and Methods

### 2.1. CC Tissues and Cells

The CC tissues were collected from the Department of Obstetrics and Gynecology, The Affiliated Jiangyin Hospital of Southeast University Medical College. All patients have signed informed consent. Human cervical cancer cell lines C33 A, Caski, Siha, and HeLa were purchased from the Cell Center of Shanghai Institutes for Biological Sciences. HaCaT cells were used as normal controls, which were obtained from Nanjing Kaiji Biotechnology Company. Cells were cultured in Dulbecco's modified Eagle's medium (Gibco, USA) supplemented with 10% fetal bovine serum (Gibco, USA) and 1% penicillin/streptomycin (Gibco, USA) at 37 °C with an atmosphere of 5% CO_2_ in a humidified cell chamber.

### 2.2. qRT-PCR and RNA Extraction

Firstly, we utilized the TRIzol reagent (Invitrogen, USA) to extract the RNA from CC cells. Then the reverse transcription process was carried out according to the instructions of PrimeScript RT Reagent (TaKaRa, Japan) and all PCRs were conducted with SYBR Premix Ex Taq Kit (TaKaRa, Japan) according to the manufacturer's instructions. *β*-Actin was used as a normal control. The 2 ^ ^ΔΔCT^ method was used to quantify the relative expression. The involved primers were as follows: *β*-actin forward, 5′'-GCATCGTCACCAACTGGGAC-3'; reverse, 5′'-ACCTGGCCGTCAGGCAGCTC-3'; DIAPH3, forward, 5′'-ACGGATGATATGCTGGACAA-3'; reverse, 5′'-CAGTGGCTTTGGAAAGTTCT-3'. The experiment was performed in triplicate.

### 2.3. CCK8 Assay

We utilized the Cell Counting Kit-8 (Dojindo, Kumamoto, Japan) to assess the proliferation ability of CC cells. Cells in different treatment groups were seeded into 96-well plates (1,000 cells/well) and cultured with complete medium for 5 days. Then the cells were incubated with CCK-8 solution for 2 h. Next, the proliferation ability was evaluated by OD value at 450 nm. Each sample was performed in triplicate.

### 2.4. Colony Formation Assay

Cells in different treatment groups were seeded in 6-well plates (500 cells/well) and placed in the incubator for 2 weeks. After that, the cells were stained with crystal violet for 30 minutes. The numbers of colony formations were counted. The experiment was performed in triplicate.

### 2.5. EdU Assay

To further assess the proliferation ability, we also utilized the EdU kit (Beyotime Biotechnology, Shanghai, China). The treated cells were firstly plated into 96-well plates (3 × 10^4^/well) and cultured for 24 hours. Then we added the EdU reagent (50 *μ*mol/L). Next, cells were permeabilized with 0.5% TritonX-100 for 10 minutes after fixing in 4% formaldehyde for 2 hours. 1×Apollo reaction solution (400 *μ*L) reacted with EdU (Beyotime Biotechnology, Shanghai, China) for 30 minutes, and DAPI (400 *μ*L) stained the nucleus after washing with PBS for three times. Finally, the microscope was used to capture the images of cells. The experiment was performed in triplicate.

### 2.6. Western Blot Assay

The concentrations of the extracted protein were evaluated by BCA kit (Beyotime Biotechnology, Shanghai, China). After electrophoresis, the PVDF membranes were used. After blocking with 5% nonfat milk at a shaker for 2 hours, we incubated the membranes with different specific primary antibodies at 4°C overnight. In another day, the membranes were incubated with the corresponding secondary antibodies for 2 hours. The antibodies were listed as follows: *β*-actin (CST, #3700, 1 : 1000), DIAPH3 (Abcam, ab227276, 1 : 1000), AKT (Abcam, ab8805, 1 : 500), p-AKT (Abcam, ab38449, 1 : 1000), mTOR (Abcam, ab134903, 1 : 10000), PTEN (Abcam, ab32199, 1 : 10000), and p-p70s6k (CST, #9208, 1 : 1000). All assays were performed in triplicate.

### 2.7. Animal Experiment

To establish the tumor growth models, we purchased four-week-old female BALB/*c* nude mice. The transfected cells (2 × 10^6^ cells/100 *μ*L) with PBS suspension were subcutaneously injected. After 5 weeks, we sacrificed the mice and observed the tumor progression.

### 2.8. Analysis of Tumor Immune Infiltrated Cells (TIICs)

We conducted the association analysis between the expression of DIAPH3 and the fractions of tumor infiltrated immune cells in CESC by using the data from the CIBERSORT and TIMER databases. The correlation was examined by Spearman test.

### 2.9. Statistical Analysis

The data were presented as mean ± SD. We statistically analyzed the data through Student's *t*-test using GraphPad Prism 7. Survival analysis was calculated by Kaplan-Meier plots. *p* values <0.05 represented a statistically significant difference. The association of DIAPH3 with clinicopathological characteristics was analyzed by the *χ*^*2*^ test.

## 3. Results

### 3.1. DIAPH3 Was Overexpressed in Cervical Cancer and Showed a Poor Prognosis

**Figure 1:**
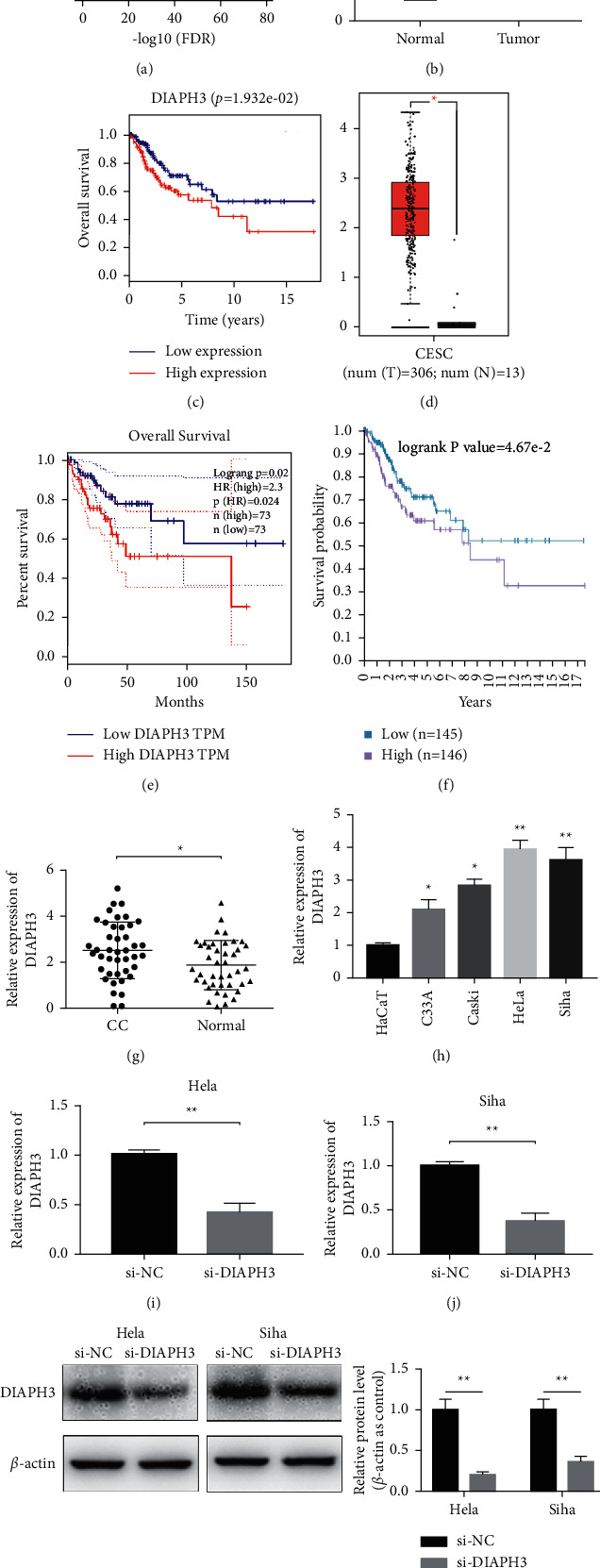
DIAPH3 was overexpressed in cervical cancer and showed a poor prognosis. (a) The volcano map of differential genes in cervical cancer in TCGA database. Fold change >2, *P* value < 0.01. (b) DIAPH3 was overexpressed in cervical cancer by analysis of TCGA data. (c) High expression of DIAPH3 displayed a poor overall survival in TCGA database. (d) The expression of DIAPH3 in cervical cancer according to GEPIA database. (e, f) The overall survival rates of DIAPH3 in GEPIA database and The Protein Atlas, respectively. (g) DIAPH3 was upregulated in CC cells compared with HaCaT. (h, i) The relative expression of DIAPH3 after transfection of si-NC and si-DIAPH3 in HeLa and Siha cells by qRT-PCR. (j, k) The relative expression of DIAPH3 after transfection of si-NC and si-DIAPH3 in HeLa and Siha cells by western blot. *β*-Actin was used as an internal control.

We first retrieved data of cervical cancer from the TCGA database, and we selected DAIPH3 as a potential oncogene in CC according to fold change >2 and *P* value < 0.01 (Figure 1(a)). To further confirm it in a large cohort of patient samples, we analyzed the expression of DIAPH3 in CC in TCGA database, consisting of 305 tumor tissues and 3 normal tissues. It showed that DIAPH3 was significantly overexpressed in CC compared with normal samples (Figure 1(b)). Moreover, we found that high expression of DIAPH3 indicated a poor overall survival in CC according to TCGA database (Figure 1(c)). Simultaneously, we verified the above results through GEPIA database and it showed the similar outcome (Figures 1(d) and 1(e)). Then we added The Human Protein Atlas and we found that patients with high expression of DIAPH3 exhibited a lower overall survival rate (Figure 1(f)). Moreover, we performed the analysis of DIAPH3 expression with survival in CC by using the TCGA-CESC cohort. Combined with the univariate cox (Table 1) and multivariate cox analysis (Table 2), it indicated that the expression of DIAPH3 was an independent prognostic risk index of cervical cancer. And we found that DIAPH3 expression was significantly upregulated in 42 pairs of CC samples compared to normal cases (Figure 1(g)). To determine the functions of DIAPH3 in CC in vitro, we examined DIAPH3 expression in four CC cells (HeLa, Siha, C33 A, and Caski) and HaCaT cells (normal control). It was shown that DIAPH3 was increased in CC cells, especially in HeLa and Siha cells (Figure 1(h)), so we chose these two cell types for further investigation. Additionally, we transfected si-NC and si-DIAPH3 into HeLa and Siha cells, respectively, and we validated the knockdown efficiency of DIAPH3 using qRT-PCR and western blot assay. We observed that the expression of DIAPH3 was obviously decreased in the si-DIAPH3 group (Figures 1(i)–1(k)). Additionally, we evaluated the association between the expression of DIAPH3 and patients' pathological characteristics. In the group of tumor size larger than 3 cm, the expression level of DIAPH3 was significantly upregulated (Table 3).

### 3.2. Knockdown of DIAPH3 Inhibited the Progression of CC In Vitro and In Vivo

**Figure 2:**
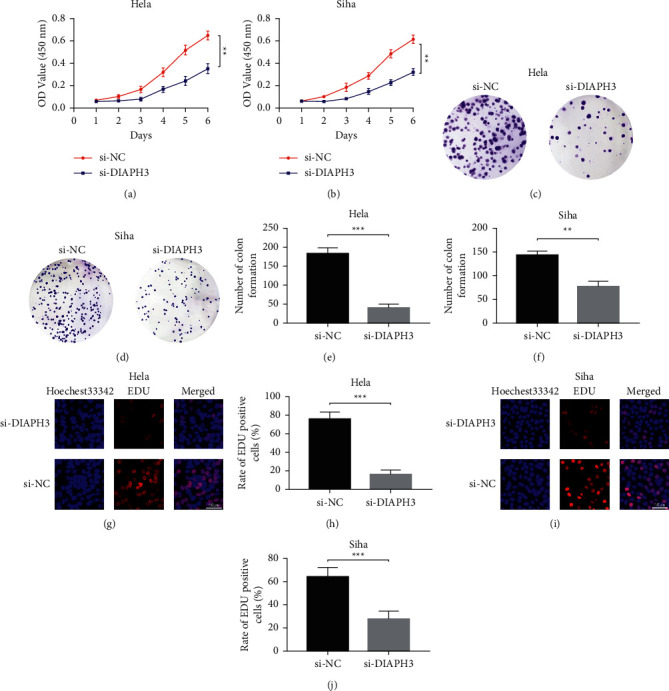
Knockdown of DIAPH3 inhibited cell proliferation in vitro. (a, b) Cell viability in si-DAIPH3 group was notably decreased compared to that in si-NC group in HeLa and Siha cells by CCK8 assay. (c, d) The representative images of colony formation assay after transfection in HeLa and Siha cells. (e, f) Knockdown of DIAPH3 decreased the number of colony formation in HeLa and Siha cells. (g, h) The rates of EdU-positive cells were declined in the si-DIAPH3 group compared with those in si-NC group in HeLa cells. (i, j) The rates of EdU-positive cells were declined in the si-DIAPH3 group compared with those in si-NC group in Siha cells.

To determine the biological functions of DIAPH3 in CC, we transfected si-NC and si-DIAPH3 into HeLa and Siha cells, respectively. After transfection, we utilized different means to detect cell proliferation in vitro. In CCK8 assay, it was shown that the ability of proliferation in si-DAIPH3 group was notably declined compared to that in si-NC group in HeLa and Siha cells (Figures 2(a) and 2(b)). In the colony formation assay, the results showed that knockdown of DIAPH3 weakened colony formation ability in HeLa cells (Figures 2(c) and 2(e)), and it showed similar results in Siha cells (Figures 2(d) and 2(f)). Then EdU incorporation assay was utilized for further assessment of the influence of DIAPH3 on CC cell proliferation. We found that the rate of EdU in the si-DIAPH3 group was obviously declined compared to that in si-NC group in HeLa cells (Figures 2(g) and 2(h)), and the tendency was consistent with that in Siha cells (Figures 2(i) and 2(j)).

**Figure 3:**
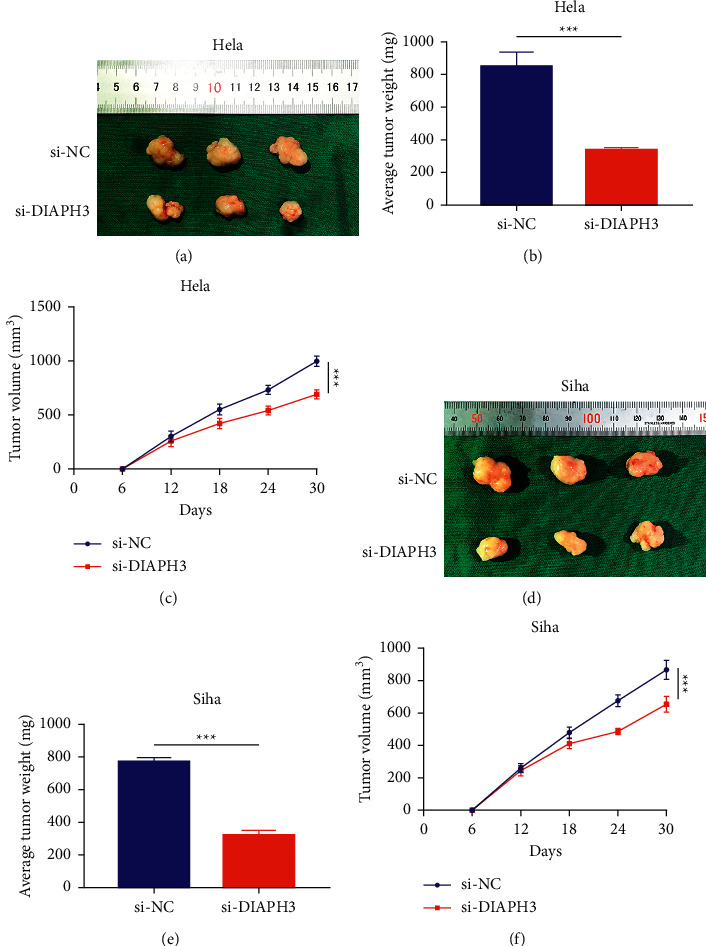
Knockdown of DIAPH3 suppressed the progression of CC in vivo. (a–c) The tumor volume and weight in the si-DiAPH3 group were obviously decreased in HeLa cells. (d–f) The tumor volume and weight in the si-DiAPH3 group were obviously decreased in Siha cells.

To further investigate the functions of DIAPH3 on tumor development, we performed xenograft assays. The transfected cells were subcutaneously injected. After 5 weeks, we sacrificed the mice and obtained the tumor. The result indicated the tumor weight and volume in the si-DiAPH3 group were obviously decreased in HeLa cells. And it exhibited similar results in Siha cells (Figures 3(d)–3(f)). Collectively, these results demonstrated that knockdown of DIAPH3 could suppress the progression of CC *in vitro* and *in vivo*.

### 3.3. Knockdown of DIAPH3 Inhibited Cell Proliferation through Suppression of mTOR Signaling Pathway

**Figure 4:**
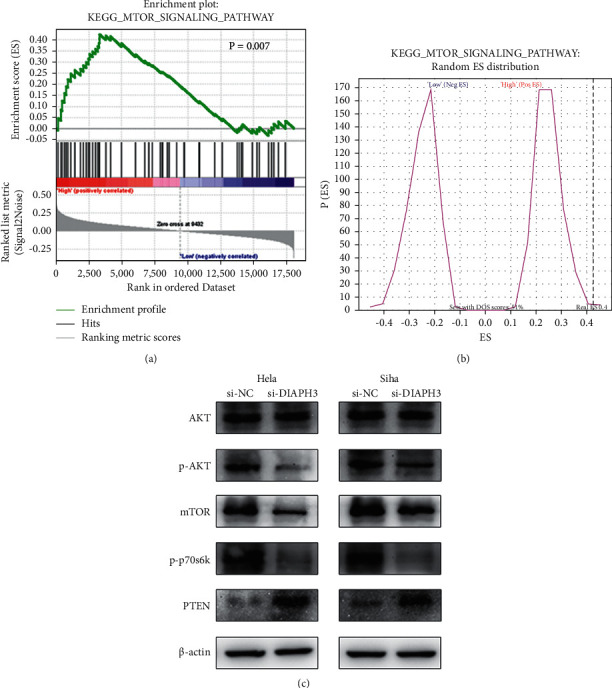
Knockdown of DIAPH3 inhibited cell proliferation through suppression of mTOR signaling pathway. (a, b) High expression of DIAPH3 was positively correlated with mTOR signaling pathway by GSEA. *P* < 0.01. (c) Knockdown of DIAPH3 decreased the expression of related proteins p-AKT, mTOR, and p-p70s6k and increased the expression of PTEN in mTOR signaling pathway in HeLa and Siha cells.

After that, we utilized bioinformatic analysis to determine the underlying mechanism of DIAPH3 on CC cell proliferation. We first analyzed bioprocess pathways by Gene Set Enrichment Analysis (GSEA). The results of Figures 4(a) and 4(b) demonstrated that high expression of DIAPH3 was positively correlated with the mTOR pathway (*P* value < 0.01). Next, we used western blot assay for further investigation. In HeLa cells, it showed that downregulated level of DIAPH3 decreased the expression of related proteins p-AKT, mTOR, and p-p70s6k and increased the expression of PTEN in mTOR signaling pathway and there is a similar trend in Siha cells (Figure 4(c)).

Accordingly, these data indicated that knockdown of DIAPH3 inhibited the proliferation of cervical cancer cells through inactivating the mTOR signaling pathway.

### 3.4. Correlation between the Expression of DIAPH3 and the Fractions of TIICs in CC

**Figure 5:**
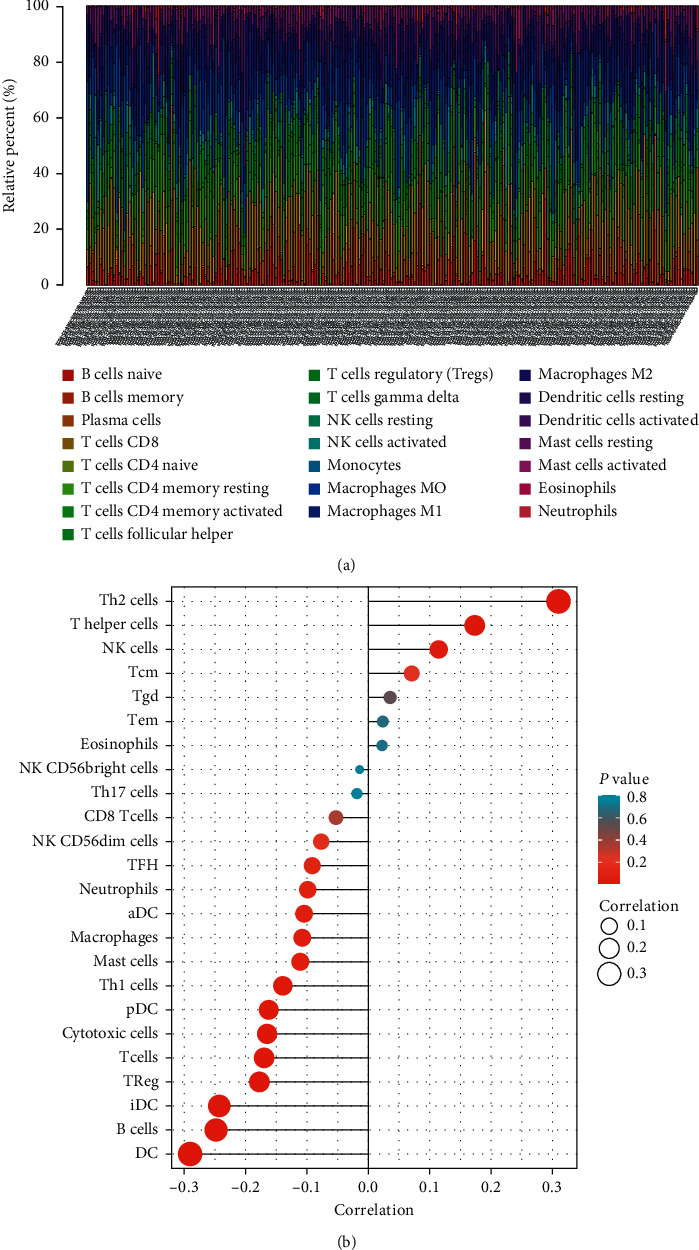
The fractions of tumor-infiltrating immune cells in CESC and correlation analysis with CIBERSORT. (a) The fractions of 22 kinds of tumor-infiltrating immune cells in CESC. (b) Association between the expression of DIAPH3 and relative abundance of 22 immune cells. The size of dots demonstrates the absolute value of Spearman R.

**Figure 6:**
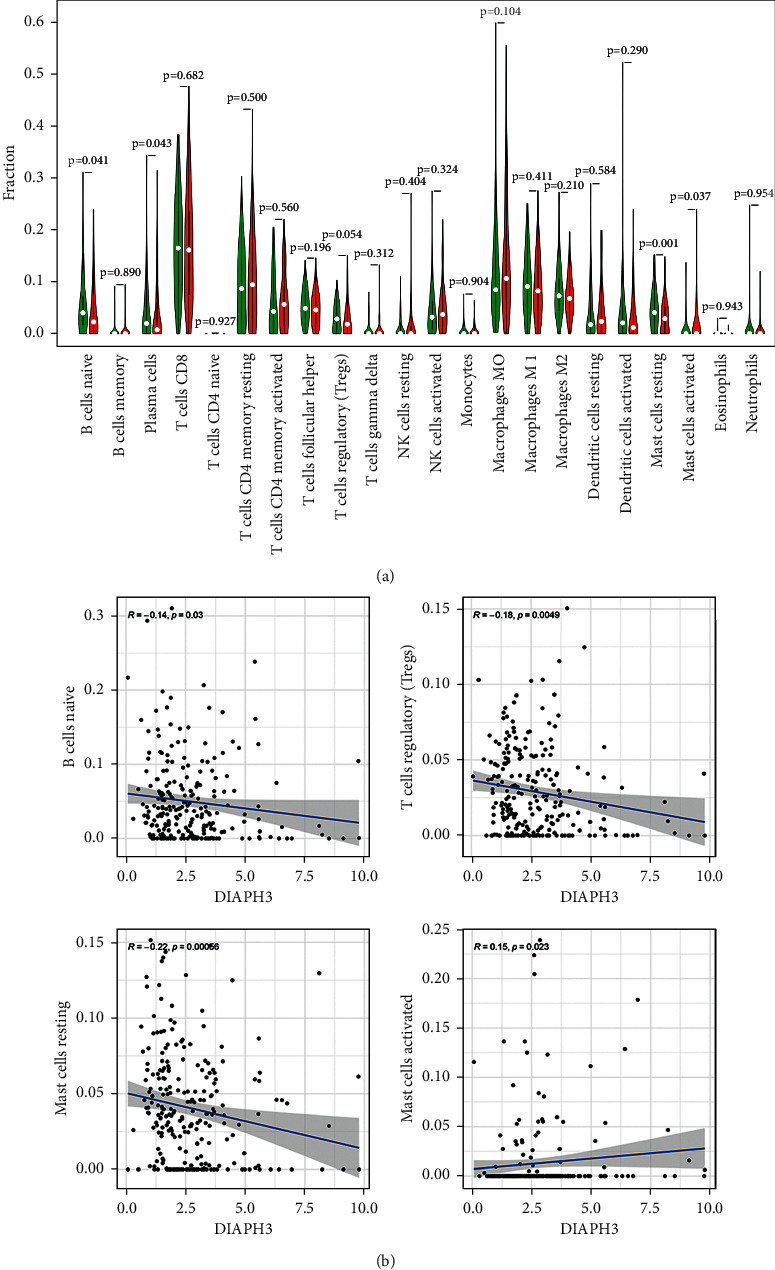
The correlation between the expression of DIAPH3 and fractions of tumor-infiltrating immune cells. (a) Comparison of 22 kinds of tumor-infiltrating immune cells between high and low DIAPH3 expression groups. The green violin diagram presents the low DIAPH3 expression group, and the red violin diagram presents the high DIAPH3 expression group. Wilcoxon rank-sum was used for the significance test. (b) The correlation between tumor-infiltrating immune cells and the expression of DIAPH3.

To further probe the association between DIAPH3 expression and TIICs in CC, we analyzed the subgroups of tumor-infiltrating immune cells by analysis of the data from CIBERSORT. Then we constructed 22 kinds of immune cell profiles (Figure 5(a)). As shown in Figure 5(b) and Table 4, the expression of DIAPH3 was positively associated with the abundance of acquired immunocytes (T helper cells (*R* = 0.173, *P*=0.002), Th2 cells (*R* = 0.310, *P* < 0.001), and NK cells (*R* = 0.115, *P*=0.045)) and negatively related to the abundance of innate immunocytes (B cells (*R* = −0.248, *P* < 0.001), Treg (*R* = −0.177, *P*=0.002), DCs (*R* = −0.290, *P* < 0.001), iDCs (*R* = −0.243, *P* < 0.001), pDCs (*R* = −0.162, *P*=0.004), cytotoxic cells (*R* = −0.165, *P*=0.004), etc.). By differential correlation analysis, we observed that three types of TIICs were negatively associated with the expression of DIAPH3, including regulatory T cells (Tregs), naïve B cells, and Mast cells resting. But Mast cells activated were shown to be positively correlated with the expression of DIAPH3 (Figures 6(a) and 6(b)).

**Figure 7:**
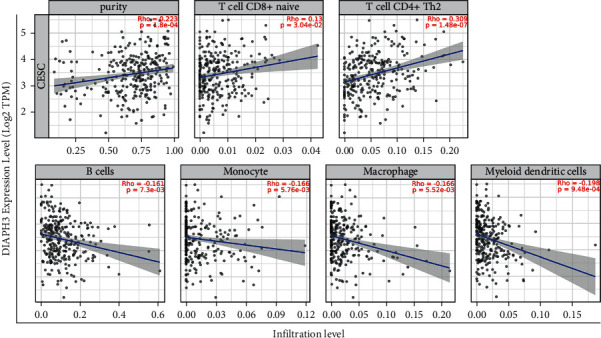
Correlation of DIAPH3 expression with immune infiltration level in CESC with TIMER database.

**Figure 8:**
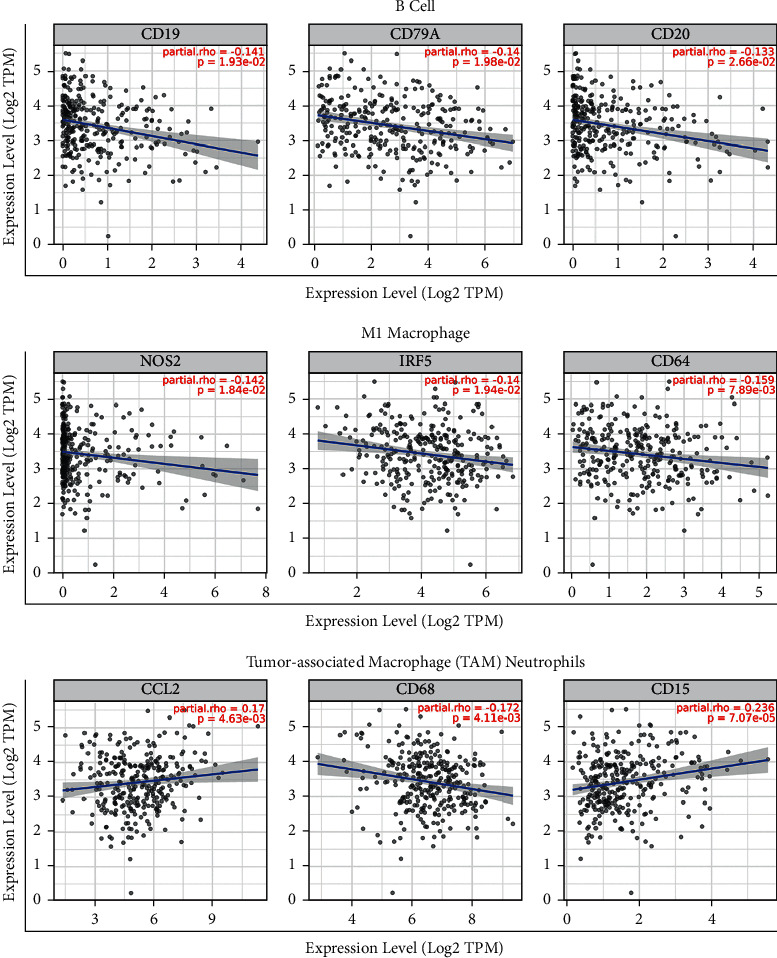
Correlation of DIAPH3 expression with marker genes of B cells, M1 macrophages, tumor-associated macrophage (TAM), and neutrophils in CESC with TIMER database.

Meanwhile, we utilized the TIMER database to further analyze the correlation between the expression of DIAPH3 and diverse kinds of TIICs.  Figure 7(a) shows that the expression of DIAPH3 was positively associated with the infiltrating levels of T cell CD8+ naive (cor = 0.13, *p* = 3.04e-02) and T cell CD4+ Th2 (cor = 0.309, *p* = 1.48e-07) and negatively related to the infiltrating levels of B cells (cor = -0.161, *p* = 7.3e-03), monocytes (cor = -0.166, *p* = 5.76e-03), macrophages (cor = -0.166, *p* = 5.52e-03), and myeloid dendritic cells (cor = -0.198, *p* = 9.48e-04). We found that DIAPH3 expression is associated with part of the markers of different immune cells (Table 5), especially negatively related to the markers of B cell and M1 macrophages (Figure 8).

### 3.5. Correlation between DIAPH3 Expression and Immune Checkpoint

**Figure 9:**
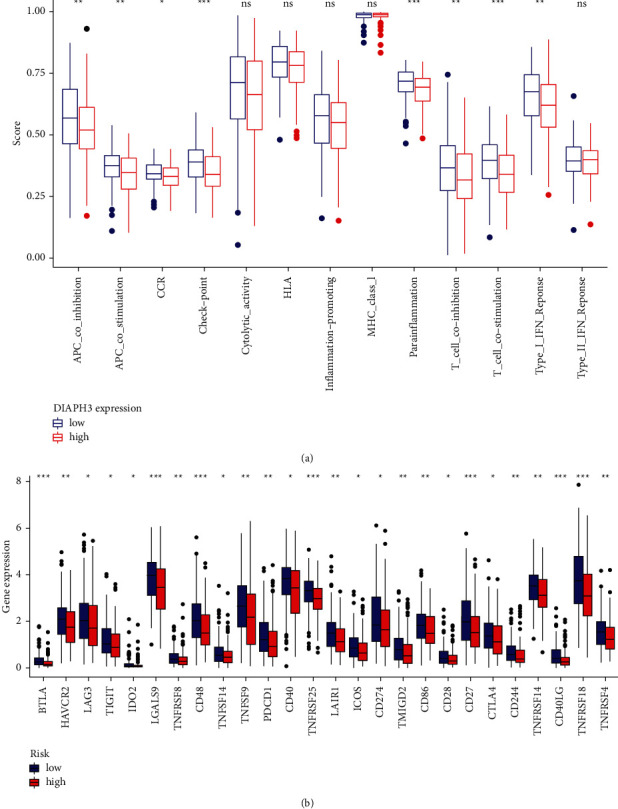
Correlation between the expression of DIAPH3 and the immune checkpoint. (a) ssGSEA for the association between immune cell subpopulations and related functions. (b) Expression of immune checkpoints among high- and low-risk DIAPH3 groups.

Firstly, we divided the expression of DIAPH3 in TCGA-CESC into high and low groups according to the median value. The results of the correlation analysis between immune cell subgroups and functions revealed that T_cell_costimulation, T_cell_coinhibition, checkpoint, APC_coinhibition, APC_costimulation, type I INF response, CCR, and parainflammation were significantly different between the low- and high-risk groups (Figure 9(a)). Simultaneously, in view of the increasing importance of immunotherapy based on checkpoint inhibitors in antitumor therapy, we probed the difference in the expression of immune checkpoints between the low-risk and high-risk groups. We found a significant difference in the expression of BTLA, CD48, PDCD-1, etc. between the low-risk and high-risk groups of patients (Figure 9(b)).

## 4. Discussion

Many researchers verified that DIAPH3 could regulate growth and migration by affecting cytoskeleton formation in different types of cancer [11, 12, 18]. And Hager MH et al. identified that DIAPH3 could accelerate the cellular transition to amoeboid tumor phenotype [19]. However, its role and molecular mechanism remain unknown in cervical cancer. We firstly reported that DIAPH3 was overexpressed in cervical cancer and enhanced the ability of CC cell proliferation. Moreover, we found that high expression of DIAPH3 indicated a poor prognosis.

The mTOR kinase is a core downstream molecule of PI3K/Akt to modulate cellular growth, metabolism, and migration, and its signaling pathway is frequently dysregulated in a variety of cancers [20–22]. Activation of the mTOR signaling pathway has been observed in cervical cancer [23, 24]. The inhibition of mTOR by rapamycin and its analogs seems to be effective in cancer treatment [25]. Importantly, therapy targeting the mTOR signaling pathway indicates clinical benefits in cervical cancer [26]. In our study, we firstly explored the notion that knockdown of DIAPH3 inhibits the proliferation of cervical cancer cells through inactivating the mTOR signaling pathway.

Recently, studies have reported that TIICs could regulate tumor progression [27]. Additionally, the poor prognosis of tumor patients is associated with the accumulation of TIICs in HCC [28]. In the current study, we revealed that the expression of DIAPH3 in CC was negatively associated with some innate immunocytes infiltration, such as B cells, DCs, and cytotoxic cells. Three new studies reported that, with the presence of the two key elements (B cells and tertiary lymphatic structure) in tumors, cancer patients will have better results when they receive immunotherapy [28–31]. DCs can significantly affect the regulation of cancer immune response [32]. Moreover, cytotoxic cells are also critical antitumor cells. A study reported that HCC cells suppressed cytotoxic T cells to modulate tumor resistance to PD1 [33]. In summary, the above results indicated that DIAPH3 plays important roles in the regulation of TIICs in CC. We hope that DIAPH3 may become a novel therapy target in cervical cancer.

## Figures and Tables

**Table 1:** Univariate Cox regression analysis of OS in TCGA-CESC cohort.

	HR	HR.95L	HR.95H	*p* value
Age	1.012642719	0.967516855	1.059873294	0.589082707
Grade	1.347682976	0.60019885	3.026079445	0.469675039
T	1.571480239	0.791716299	3.1192362	0.196265045
N	1.926434151	0.732327039	5.067610971	0.183955862
DIAPH3	1.46513653	1.182831075	1.814819628	0.000469571^*∗∗∗*^

^*∗∗∗*^ *p* < 0.001 statistically significant difference.

**Table 2:** Multivariate Cox regression analysis of OS in TCGA-CESC cohort.

	HR	HR.95L	HR.95H	*p* value
Age	0.997843949	0.952416863	1.045437753	0.927658286
Grade	1.045822636	0.461258359	2.371219872	0.914572354
T	1.367948739	0.698662983	2.678378272	0.360744139
N	2.418271676	0.852581947	6.859209164	0.096887932
DIAPH3	1.518865321	1.184673829	1.947330823	0.000978608^*∗∗∗*^

^*∗∗∗*^ *p* < 0.001 statistically significant difference.

**Table 3:** Expression of DIAPH3 in cervical cancer according to patients' clinicopathological characteristics.

Characteristics	Number	DIAPH3 expression		*P* value
		High-risk group	Low-risk group	
Age (years)				
<50	19	11	8	0.352
≥50	23	10	13	

Differentiation				
Well/moderate	26	12	14	0.525
Poor	16	9	7	

Size (cm)				
<3	24	8	16	0.013^*∗*^
≥3	18	13	5	

FIGO stage				
I	24	9	15	0.061
II and III	18	12	6	

Lymph node metastasis				
No	25	13	12	0.753
Yes	17	8	9	

^*∗*^ *p* < 0.05 statistically significant difference.

**Table 4:** Association between expression of DIAPH3 and immune infiltration.

Gene	Cells	Correlation (Spearman)	*P* value (Spearman)
DIAPH3	aDC	−0.104	0.068
DIAPH3	B cells	−0.248	<0.001
DIAPH3	CD8 T cells	−0.053	0.357
DIAPH3	Cytotoxic cells	−0.165	0.004
DIAPH3	DC	−0.290	<0.001
DIAPH3	Eosinophils	0.022	0.699
DIAPH3	iDC	−0.243	<0.001
DIAPH3	Macrophages	−0.108	0.060
DIAPH3	Mast cells	−0.111	0.053
DIAPH3	Neutrophils	−0.099	0.085
DIAPH3	NK CD56bright cells	−0.014	0.807
DIAPH3	NK CD56dim cells	−0.077	0.179
DIAPH3	NK cells	0.115	0.045
DIAPH3	pDC	−0.162	0.004
DIAPH3	T cells	−0.170	0.003
DIAPH3	T helper cells	0.173	0.002
DIAPH3	Tcm	0.071	0.218
DIAPH3	Tem	0.024	0.680
DIAPH3	TFH	−0.091	0.112
DIAPH3	Tgd	0.036	0.536
DIAPH3	Th1 cells	−0.139	0.015
DIAPH3	Th17 cells	−0.019	0.746
DIAPH3	Th2 cells	0.310	<0.001
DIAPH3	TReg	−0.177	0.002

**Table 5:** Correlation analysis between DIAPH3 expression and related markers of immune cells using data in TIMER database.

Description	Gene markers	CESC	P
		Cor	
CD8+ T cell	CD8A	0.013	0.835
	CD8B	0.091	0.129
	CD45	0.027	0.649

T cell (general)	CD3D	0.114	0.058
	CD3E	0.098	0.104
	CD2	0.104	0.0827

B cell	CD19	0.141	0.0193^*∗*^
	CD79 A	-0.14	0.0198^*∗*^
	CD27	0.112	0.0635
	CD20	0.113	0.0266^*∗*^

Monocyte	CD14	0.092	0.128
	CD115 (CSF1R)	0.044	0.465

TAM	CCL2	0.17	0.00463^*∗∗*^
	CD68	0.172	0.00411^*∗∗*^
	IL10	0.032	0.596

M1 macrophage	INOS (NOS2)	0.142	0.0184^*∗*^
	CD80	0.022	0.717
	IRF5	−0.14	0.0194^*∗*^
	IL6	0.192	0.00134^*∗∗*^
	CD64 (FCGR1A)	0.159	0.00789^*∗∗*^

M2 macrophage	CD163	−0.05	0.407
	CD206	0.011	0.853
	VSIG4	0.098	0.105
	MS4A4A	0.055	0.362

Neutrophils	CD66b (CEACAM8)	0.013	0.825
	CD11b (ITGAM)	0.056	0.357
	CD15	0.236	0.0000707^*∗∗∗*^

Natural killer cell	KIR2DL1	0.05	0.406
	KIR2DL3	0.025	0.678
	KIR3DL1	0.004	0.953
	KIR3DL2	0.01	0.873
	CD56	0.009	0.884
	CD335 (NKp46)	0.004	0.942

Dendritic cell	BDCA-1 (CD1C)	0.061	0.316
	BDCA-3 (CD141)	−0.05	0.41
	BDCA-4 (NRP1)	0.036	0.549
	CD123	0.045	0.453
	CD11c (ITGAX)	0.083	0.168

Th1	T-bet (TBX21)	0.071	0.237
	STAT4	−0.005	0.931
	STAT1	0.017	0.78

Th2	GATA3	0.068	0.26
	STAT6	0.011	0.857
	IL13	0.025	0.68

Tfh	BCL6	0.064	0.285
	IL21	0.066	0.275

Th17	STAT3	0.028	0.645
	IL17A	0.018	0.767

Treg	FOXP3	0.019	0.75
	CD25	0.014	0.813
	CCR8	0.014	0.821
	STAT5B	0.066	0.274

T cell exhaustion	PD-1 (PDCD1)	0.064	0.289
	CTLA4	0.035	0.567
	LAG3	0.036	0.548
	TIM-3 (HAVCR2)	0.092	0.128

^*∗*^ *p* < 0.05; ^*∗∗*^*p* < 0.01; ^*∗∗∗*^*p* < 0.001.

## Data Availability

The data used to support the findings of this study are available from the corresponding author upon request.
